# Is Plant Life-History of Biseasonal Germination Consistent in Response to Extreme Precipitation?

**DOI:** 10.3390/plants10081642

**Published:** 2021-08-10

**Authors:** Yanfeng Chen, Hui Zhang, Lingwei Zhang, Lan Zhang, Qiumei Cao, Huiliang Liu, Daoyuan Zhang

**Affiliations:** 1 State Key Laboratory of Desert and Oasis Ecology, Xinjiang Institute of Ecology and Geography, Chinese Academy of Sciences, Urumqi 830011, China; chenyanfeng@qfnu.edu.cn (Y.C.); Z13150306508@163.com (L.Z.); caoqiumei@ms.xjb.ac.cn (Q.C.); Zhangdy@ms.xjb.ac.cn (D.Z.); 2 Department of Tourism Management, School of Geography and Tourism, Qufu Normal University, Rizhao 276826, China; 3 Department of Information Engineering, Shandong Water Polytechnic, Rizhao 276826, China; zhanghui@shandong.cn; 4 Xinjiang Key Laboratory of Soil and Plant Ecological Processes, College of Grassland and Environment Sciences, Xinjiang Agricultural University, Urumqi 830052, China; zlwlz@163.com; 5 College of Resources and Environment, University of Chinese Academy of Sciences, Beijing 100049, China; 6 Yili Botanical Garden, Xinjiang Institute of Ecology and Geography, Xinyuan 835800, China; 7 Turpan Eremophytes Botanical Garden, Chinese Academy of Sciences, Turpan 838008, China

**Keywords:** climate change, ephemeral plant, *Erodium oxyrhynchum*, extreme precipitation, plant growth

## Abstract

Future climate is projected to increase in the intensity and frequency of extreme precipitation events, and the resulting ecological consequences are often more serious than those of normal precipitation events. In particular, in desert ecosystems, due to the low frequency and strong fluctuation of extreme precipitation, the destructive consequences for desert plants caused by extreme precipitation have not received enough attention for some time. Based on statistics of extreme precipitation events (1965–2018) in the Gurbantunggut Desert, we investigated the effects of extreme precipitation (+0%, CK; +50%, W1; +100%, W2; +200%, W3; maintenance of field capacity, W4) on the plant life-history of the spring-germinated (SG) and autumn-germinated (AG) ephemeral plant *Erodium oxyrhynchum* by monitoring seedling emergence, survival, phenology, organ size, biomass accumulation, and allocation. The results showed that extreme precipitation caused about 2.5% seedling emergence of *E. oxyrhynchum* in autumn 2018 and 3.0% seedling emergence in early spring 2019, which means that most seeds may be stored in the soil or have died. Meanwhile, extreme precipitation significantly improved the survival, organ size, and biomass accumulation of SG and AG plants, and W3 was close to the precipitation threshold of SG (326.70 mm) and AG (560.10 mm) plants corresponding to the maximum individual biomass; thus, AG plants with a longer life cycle need more water for growth. Conversely, W4 caused AG plants to enter the leaf stage in advance and led to death in winter, which indicates that extreme precipitation may not be good for AG plants. Root and reproduction biomass allocation of SG and AG plants showed a significantly opposite trend under extreme precipitation treatments, which might be related to their different life-history strategies. Therefore, when only taking into account the changing trend of extreme precipitation from the Coupled Model Intercomparison Project 6 (CMIP6) climate projections data, we speculate that extreme precipitation may promote the growth of SG and AG plants from the beginning to the middle of this century, but extreme precipitation in autumn exceeding a certain threshold may adversely affect the survival of AG plants at the end of the century.

## 1. Introduction

Extreme precipitation is generally defined as precipitation that is characterized by larger individual events and extended periods of time between events, and usually use a certain percentile value as the extreme precipitation threshold, such as the 95th percentile [1]. Meanwhile, extreme precipitation events often cause dramatically more fluctuations in soil water content than those of normal precipitation events, especially since extreme precipitation exceeding a certain threshold often pushes plants to their physiological limits, from which function cannot recover or even results in plant death [2,3,4]. Therefore, objectively choosing realistic extremeness and describing extremeness in relation to local conditions are crucial to the identification of precipitation threshold.

Generally, larger or more frequent extreme precipitation events often result in constant or temporal waterlogging and hypoxia in root systems, which further restricts the life activity of plants and makes it difficult for them to grow. For example, research on seedlings of silver birch (*Betula pendula* Roth) suggested that waterlogging caused by long-term extreme precipitation promoted the formation of thin roots and increased the high density of non-glandular trichomes and leaves, which suggests an adaptive mechanism with respect to excess soil water [5]. Similarly, due to impaired functions of root systems, waterlogging also reduced accumulation and remobilization of carbohydrates into grains, grain yield production, and grain quality of wheat [6]. Furthermore, in the wetland ecosystem of China, flooding not only affected the reproduction phenology of *Sinojackia huangmeiensis* but could also respond to extreme precipitation-induced waterlogging by changing both seed morphological traits and element concentrations [7]. Conversely, in the grassland ecosystem of the Great Plains, short-term extreme precipitation events resulted in a 30% increase in above-ground net primary productivity at the semiarid steppe [8]. In South African savanna, simulation experiments showed that the population dynamics of wood plants (*Grewia flava*) also demonstrated a positive trend in the scenario of an increase in extreme precipitation by 30–40% and a negative trend for a decrease by 5–15% [9]. Therefore, extreme precipitation events can affect plant growth and population dynamics due to the differences in initial site conditions, such as the aridity index, soil type, and plant functional types, and the influence of extreme precipitation events on plants is not consistent across ecosystems.

Studies suggest that water-limited ecosystems are generally associated with positive responses of plants to extreme precipitation, mainly because the soil moisture content in arid areas is inherently low, the drought index is small, and extreme precipitation can effectively supple soil moisture, as it remains below the threshold of maximum soil moisture [10,11,12]. For example, within ecosystems that are water limited (<600 mm mean annual precipitation, or an aridity index < 1.0), extreme precipitation increases soil water content and above-ground net primary productivity [13]. In contrast, in ecosystems that are not water limited (>600 mm mean annual precipitation, or an aridity index > 1.0), extreme precipitation leads to flooding or waterlogging and is followed by a reduction in plant growth or above-ground net primary productivity [13]. Meanwhile, according to the “soil water bucket” model proposed by Gordon and Famiglietti [10], where the soil water bucket is usually low (a nearly empty bucket system) in arid ecosystems, desert plants have evolved a variety of strategies to adapt to low soil moisture environments, for example, increasing their root length to expand the root absorption area and leaves becoming smaller to reduce transpiration [14,15]. However, there is a lack of information regarding how characteristics such as roots and leaves will change when desert plants suffer short and drastic changes in soil moisture caused by extreme precipitation. Therefore, research on these issues is necessary to improve our understanding of the impact of extreme precipitation on population persistence, community stability, and ecosystem function in arid ecosystems.

The Gurbantunggut Desert is located in northwestern China and is a typical temperate desert in Central Asia. Recent studies have shown that the frequency and magnitude of extreme precipitation in the Gurbantunggut Desert have increased and often manifest strong fluctuation in seasonal and interannual precipitation [16]; especially when extreme precipitation fluctuations exceed a certain threshold, which is likely to pose a threat to population persistence of desert plants. Ephemeral plants are an important plant layer in the early spring of the Gurbantunggut Desert, and they play an important role in controlling soil wind erosion and improving the ecological environment around this desert [17,18]. Under the background of climate change, scholars have carried out extensive research regarding the impact of precipitation changes on nutrient absorption [19], physiology [20], morphology [21], life-history [17,22], population distribution [23], and community composition [24] of ephemeral plants, but research on the effects of extreme precipitation has not been carried out. Notably, *Erodium oxyrhynchum* (Geraniaceae) is the dominant herb ephemeral plant during the early spring in the Gurbantunggut Desert [25,26], and it can germinate both in spring and autumn in some years [17,18]. Spring-germinated (SG) plants complete their life cycle in about two months, before the onset of summer (spring ephemeral). Autumn-germinated plants (AG) overwinter as rosettes and complete their life cycle in the following spring (winter annual), with their life cycle being about six months in length [22]. Meanwhile, in a field site in the Gurbantunggut Desert, spring (annual) ephemeral species with short life cycles were found to be more sensitive to nitrogen deposition than annual species with long life cycles [27]. Therefore, based on the aridity index in arid regions (a nearly-empty bucket system), the differences in SG and AG plants of *E. oxyrhynchum* in the life cycle and the trends of extreme precipitation in the future, we propose the following hypothesis: (1) extreme precipitation will improve the growth of SG and AG plants and expand the population distribution of *E. oxyrhynchum*; (2) SG plants with shorter life cycles are more sensitive to extreme precipitation than AG plants. Moreover, taking into account the trend of extreme precipitation in the future, we used extreme precipitation data obtained by climate models to predict the population dynamics of *E. oxyrhynchum*. To test these hypotheses, we monitored seedling emergence, phenological transitions, the growth process, biomass accumulation, and allocation of SG and AG plants with extreme precipitation in the habitat of ephemeral plants (the Gurbantunggut Desert) for two years, as well as provided a prediction regarding the effect of future precipitation change on population dynamics. Assessing the responses of dominant species will permit powerful inferences to be made regarding the long-term implications of extreme precipitation at scales relevant to policymakers.

## 2. Results

### 2.1. The Effects of Extreme Precipitation on Seedling Emergence

**Figure 1:**
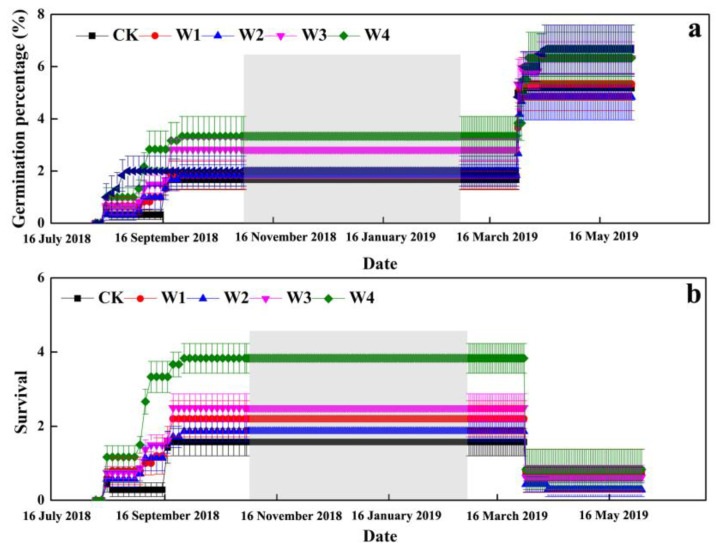
Effects of extreme precipitation on the seedling emergence (**a**) and survival percentage (**b**) of *Erodium oxyrhynchum*. CK, control treatment; W1, +30% extreme precipitation; W2, +100% extreme precipitation; W3, +200% extreme precipitation; W4, maintenance of field capacity by increasing precipitation every three days; Shaded part indicates snowfall in winter and observations were missing.

During their life-history, the seedling emergence of *E. oxyrhynchum* showed two obvious peak periods, from the end of July to the beginning of September in 2018 and from the end of March to the beginning of April in 2019 (Figure 1a). The first peak period of seedling emergence increased slowly, lasting about two months, and the seedling emergence percentage reached about 2.5%. The second peak period of seedling emergence increased rapidly with the cumulative emergence percentage reaching about 5.5% (Figure 1a), but no significant difference between W1, W2, W3, and W4 treatments was observed.

### 2.2. The Effects of Extreme Precipitation on Survival

SG plants did not show death in the whole life-history; therefore, we only counted the survival of AG plants. At the beginning of September 2018, the seedling emergence of AG plants reached the maximum in W1, W2, W3, and W4 treatments, and the effect of W3 was significant. Meanwhile, at the end of March 2019, the survival of AG plants showed a declining trend in all treatments, especially in W4 (Figure 1b).

### 2.3. The Effects of Extreme Precipitation on Phenology

The life cycle of AG plants was longer than that of SG plants, and dates of emergence and the leafing stage of AG plants occurred earlier than SG plants, but the flowering and fruiting stages of AG plants were similar to those of SG plants (Table 1). Contrarily, the withered yellow stage of SG plants occurred two days earlier than in AG plants. W1, W2, W3, and W4 delayed flowering and withering of AG and SG plants by about three to five days, with almost no effect on the fruiting stage of SG plants, and W1, W2, W3, and W4 unusually advanced the fruiting stage of AG plants (Table 1).

### 2.4. The Effects of Extreme Precipitation on Organ Size

**Figure 2:**
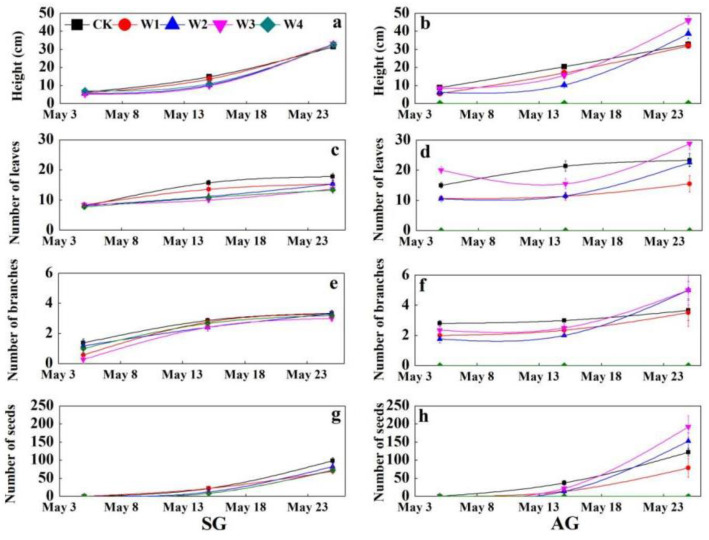
Effects of extreme precipitation on height (**a**,**b**), leaf number (**c**,**d**), branch number (**e**,**f**), and seed number (**g**,**h**) of spring-germinated (SG) plants and autumn-germinated (AG) plants of *Erodium oxyrhynchum*. (**a**), Plant height of SG; (**b**), plant height of AG; (**c**), leaf number of SG; (**d**), leaf number of AG; (**e**), branch number of SG; (**f**), branch number of AG; (**g**), seed number of SG; (**h**), seed number of AG. CK, control treatment; W1, +30% increase in precipitation; W2, +100% increase in precipitation; W3, +200% increase in precipitation; W4, maintenance of field capacity by increasing precipitation every three days.

In the control, plant height, as well as leaf, branch, and seed number of SG and AG plants showed an increasing trend during the life-history, and these traits differed significantly between SG and AG plants at the reproductive stage, i.e., AG > SG (Figure 2a–h). Meanwhile, precipitation treatments (W1, W2, W3, and W4) had no significant effect on the same traits for SG plants, but triggered variations for AG plants, especially in the flowering and fruiting stages (Figure 2a–h). Moreover, the leaf and branch number showed an increasing trend with W1, W2, W3, and W4 treatments (Figure 2b,d,f,h).

### 2.5. The Effects of Extreme Precipitation on Dry Biomass Accumulation and Allocation

**Figure 3:**
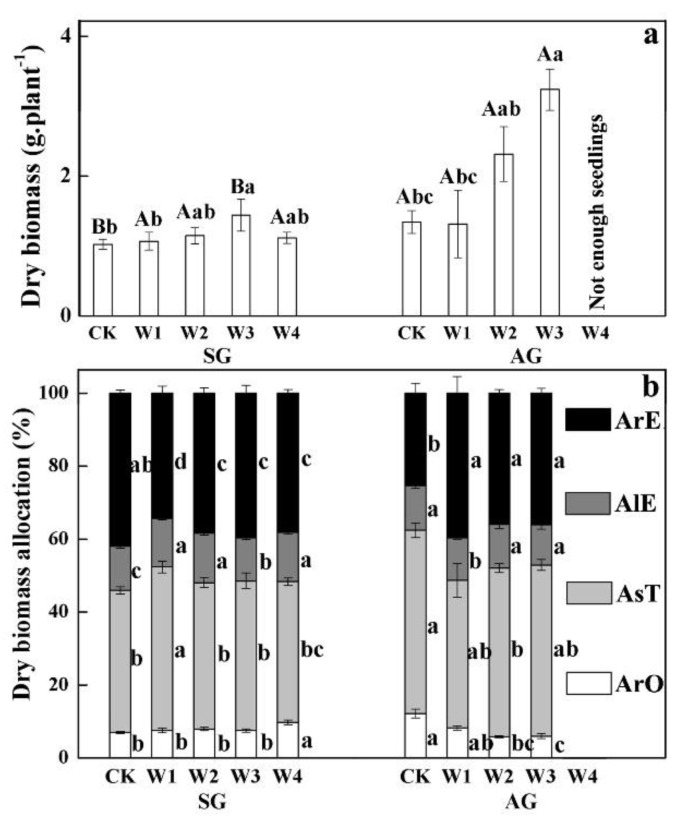
Effects of extreme precipitation on dry biomass accumulation (**a**) and biomass allocation (**b**) of spring-germinated (SG) and autumn-germinated (AG) plants of *Erodium oxyrhynchum*. ArO, allocation of biomass to roots; AsT, allocation of biomass to stems; AlE, allocation of biomass to leaves; ArE, allocation of biomass to reproductive organ. CK, control treatment; W1, +30% extreme precipitation; W2, +100% extreme precipitation; W3, +200% extreme precipitation; W4, maintenance of field capacity by increasing precipitation every three days. Different lowercase letters indicate significant differences (*p* < 0.05) among CK, W1, W2, W3, and W4 for SG and AG, and different uppercase letters significant differences between SG and AG for the same extreme precipitation treatment. Moreover, W4 treatment did not have enough seedlings for statistical analysis.

In the control, the dry biomass of AG plants was 1.31 times that of SG plants, i.e., 1.024 and 1.343 g per plant, respectively. W1, W2, W3, and W4 also had a significant effect on the biomass accumulation of SG and AG plants (*p* < 0.05, Table 2), and W3 treatment caused the highest individual biomass accumulation in SG and AG plants (Figure 3a).

For biomass allocation, W1, W2, W3, and W4 also had a significant effect on root, stem, leaf, and reproductive biomass accumulation and allocation of SG and AG plants (*p* < 0.05, Table 2). Notably, the root biomass allocation of SG plants showed an increasing trend with W1, W2, W3, and W4 treatments, while the reproductive biomass allocation of SG plants showed a decreasing trend (Figure 3b). For AG plants, the root biomass allocation showed a significant decreasing trend with W1, W2, W3, and W4 treatments, but the reproductive biomass allocation of AG plants showed an increasing trend (Figure 3b).

## 3. Discussion

Climate change is projected to increase the intensity and frequency of extreme precipitation events at high latitudes and result in greater annual precipitation variability [1]. Our results show that extreme precipitation significantly improved seedling emergence of SG and AG plants, but unexpectedly, that W4 has a negative impact on the survival of AG plants. Meanwhile, extreme precipitation delayed the reproductive phenology and improved organ size and biomass accumulation of SG and AG plants, but root and reproduction biomass allocation of SG and AG plants showed an opposite trend. Thus, our results not only determined the precipitation threshold of SG and AG plants corresponding to the maximum individual biomass for the first time, but also found different life-history strategies of SG and AG plants.

Indoor experiment results showed that seeds of *E. oxyrhynchum* have a high hardness rate and low seed coat permeability, and manual scarification effectively broke seed dormancy, with the germination percentage reaching above 90%; thus, seeds of *E. oxyrhynchum* have physical dormancy [27]. In the field, seeds of *E. oxyrhynchum* showed two obvious peak periods of seedling emergence, and the emergence percentage of the first peak period increased slowly, lasting for about two months (emergence percentage reached about 2.5%), which indicates that dry–hot treatment in summer is the main factor affecting the dormancy break of *E. oxyrhynchum*, but extreme precipitation did not significantly improve dormancy break and seedling emergence. Similarly, Buhailiqiemu [28] investigated the effects of temperature and humidity treatments on physical dormancy release of *Eremosparton songoricum*, *Ammodendron bifolium*, and *Glycyrrhiza uralensis* in the Gurbantunggut Desert, and concluded that dry–hot treatment significantly affected the breaking of physical dormancy. Thus, our results once again confirm that dry–hot treatment is important to break the physical dormancy of desert plant seeds. The second peak period indicated that low temperature in winter can also break physical dormancy with the cumulative emergence percentage reaching about 5.5%, which is in contrast with the results that suggest that cold stratification has no significant effect on physical dormancy release [28]. In the Gurbantunggut Desert, seeds not only experienced a cold stratification period but also experienced two freeze–thaw periods (before winter and when the snow melted in early spring); thus, we speculated that freeze–thaw period (cold–wet) can play a role in releasing physical dormancy of *E. oxyrhynchum*. Meanwhile, McDonald [29] found that wet treatment in the field can also quickly relieve physical dormancy in seeds of the tropical leguminous plant *Parkinsonia aculeate*, and physical dormancy of *Silene diclinis* is positively correlated with temperature and relative humidity [30]. Therefore, the results of the cumulative emergence percentage indicate that only a small percentage of the seeds harvested that year would germinate that year, most seeds may be stored in the soil by the seed bank or have died, and extreme precipitation has no significant effect on the seedling emergence of *E. oxyrhynchum*.

After seedling emergence in late autumn, seedlings inevitably suffer low-temperature stress in winter and freeze–thaw in early spring [18,22]. Especially for AG plants, at the beginning of September 2018, the cumulative emergence percentage of AG plants reached the highest value in all extreme precipitation treatments, but the survival of AG plants showed a significant declining trend at the end of March 2019, and then all AG plants died under W4 treatment in early spring of 2019. On one hand, we speculated that more water often results in constant or temporal waterlogging and hypoxia in root systems, which further restricts the life activity of plants and makes it difficult for them to grow in winter. On the other hand, AG plants germinated earlier are likely to lose their cold resistance to low temperature in winter, resulting in higher mortality rates in the leaf stage than that in the cotyledon stage. Furthermore, extreme precipitation also delayed the flowering and yellowing stage of AG and SG plants but advanced the fruiting stage of AG plants. Therefore, in the context of extreme climate change, precipitation in late autumn, winter and early spring exceeding a certain threshold may have a negative impact on survival of AG plants, but extreme precipitation in spring is beneficial to prolonging the fruiting stage in order to increase reproductive output.

Organ sizes of AG plants were significantly larger than those of SG plants, which is very similar with the results of AG and SG plants in the Gurbantunggut Desert [18,22,31]. This is mainly because AG plants have an earlier emergence phenology and longer growth cycle than those of SG plants, and they accumulate more biomass for growth and reproduction. In the flowering and fruiting stages, extreme precipitation improves plant height, root length, leaf number, and branch and seed number of SG and AG plants, which is inconsistent with the notion that extreme flooding can cause hypoxia in the rhizosphere and even death in temperate grasslands [8,32], which is likely to be related to the lower soil moisture of arid ecosystems than that of forest ecosystems [33]. Similarly, for biomass accumulation, almost extreme precipitation treatments promoted individual biomass accumulation of AG and SG plants, and W3 treatment caused the most individual biomass accumulation in SG and AG plants. Meanwhile, according to the “soil water bucket” model [10], in arid and semi-arid ecosystems in which soil water content is usually low (i.e., a nearly empty bucket system with low precipitation inputs and high evaporative demand), CK, W1, and W2 treatments increase soil water above stress levels for substantial periods of time, creating suitable conditions for plant growth. W3 treatment is close to the critical thresholds of SG and AG plants: 326.70 and 560.10 mm, respectively. On the contrary, W4 treatment (maintenance of field capacity) more completely recharges the water bucket, increasing the amount of time when soil water content is above the most optimal thresholds, which may lead to root hypoxia and inhibition of ATP synthesis and affect survival [13]. Thus, there will be a positive relationship between changes in soil water content and growth rates of the plant, with pronounced thresholds separating the stressed and unstressed states as soil water content varies. The effect of extreme precipitation on AG plants was more significant than that on SG plants, but SG plants showed a more obvious growth plasticity response to increased precipitation than that of AG in an experiment of +30% and +50% increased precipitation [22]. The significant difference is probably related to the earlier emergence phenology and longer life cycle caused by extreme precipitation. In this study, AG plants emerged in mid-August and quickly commenced the leafing stage after emergence, but AG plants in Chen’s experiment overwintered in the form of cotyledons after emergence, and the leafing stage did not occur until the second spring [34]. Therefore, AG plants in this study were significantly higher in water use efficiency than were AG plants in Chen’s experiment. In addition to biomass accumulation, root and reproduction biomass allocation of SG and AG plants showed a significantly negative correlation, which indicates that they had different life-history strategies. When precipitation and environmental conditions improve, SG plants with short life-history may initially transfer more biomass to the underground in order to absorb water and nutrients. However, in AG plants, the root grows in autumn of the previous year (Figure 2), and soil moisture can be quickly transported to the ground through the root system for vegetative and reproductive growth. Therefore, if the intensity and frequency of extreme precipitation further increase in future, AG plants may exhibit higher productivity, whereas SG plants may exhibit higher reproductive efficiency.

Based on the analysis of future precipitation trends in the SSP1 1.9, SSP2 4.5, and SSP3 8.5 scenarios, precipitation in spring and autumn shows stronger fluctuation than precipitation in summer and winter, and the trend of precipitation in spring and autumn can be divided into two obvious periods: the relatively stable period from 2020 to 2070, and the severe fluctuation period from 2070 to 2100 (Figure S1). Thus, we predict that the relatively stable period may promote the emergence of SG and AG plants equally, but the drastic fluctuations of precipitation in spring and autumn at the end of this century are likely to affect the emergence of SG and AG plants unequally, resulting in only SG plants or only AG plants in some years. However, the strategies of biseasonal emergence, which are not likely to have an effect on the population of *E. oxyrhinchum*, should be considered. Except for precipitation fluctuations of spring and autumn, we must consider the magnitude of extreme precipitation in spring and autumn. Spring precipitation predictions hardly exceed twice the current average values, but autumn ones are calculated to be even three times above them by the end of this century. Thus, combined with the results of the extreme precipitation experiment in this study, firstly, increases in spring precipitation may continue to promote plant growth within a foreseeable range, but when precipitation in autumn exceeds three times the average precipitation, it is likely to induce AG plants to enter the leaf development stage earlier, leading to freezing death from low temperature in winter. Therefore, we speculate that precipitation in spring will always promote the growth of SG and AG plants, but the drastic fluctuation of extreme precipitation in autumn is likely to cause the death of AG plants and affect the continuation of the population.

In conclusion, precipitation is mainly an environmental factor regulating the emergence of SG and AG plants in the Gurbantunggut Desert, and extreme precipitation promotes few seeds to germinate in autumn and spring and then shows a bet-hedging strategy. In essence, a bet-hedging strategy would reduce the risk of population extinction caused by one-time emergence, which is of great significance for maintaining plant diversity in desert areas. However, when precipitation exceeds a certain threshold, which often causes AG plants to the enter the leaf stage in advance, AG plants die. Meanwhile, extreme precipitation also significantly affects reproduction phenology and biomass accumulation of SG and AG plants, and W3 treatment is close to the precipitation threshold corresponding to the maximum individual biomass. For biomass allocation, root and reproduction biomass allocation of SG and AG plants shows a significantly opposite trend with an increase in extreme precipitation. Therefore, in the context of precipitation changes, extreme precipitation below the bucket threshold of arid ecosystems will promote the growth of SG and AG plants, but when extreme precipitation exceeds the bucket threshold in autumn, it is likely to cause the death of AG plants.

## 4. Materials and Methods

### 4.1. Plant Species and Study Area

*Erodium oxyrhynchum* is an annual, desert herb, and it is mainly grown in gravel Gobi, semi-fixed dunes and gullies in the piedmont zone. It is widely distributed in northwest China, as well as Kazakhstan, Caucasus, and West Asia [22]. In particular, in the Gurbantunggut Desert of northwest China, *E. oxyrhynchum* is the most common and dominant herb among the ephemeral plants [17]. In the Gurbantunggut Desert, the mean annual air temperature of the Gurbantunggut Desert is 6.6 °C, and the extreme high and low air temperatures are 42.6 (July) and −41.6 °C (January), respectively. Mean annual precipitation varies from 70 to 180 mm, and mean annual evaporation is about 2000 mm [35].

### 4.2. Climate Data (1951–2018) and Predictions of Climate Change Models (2020–2100)

Based on monthly precipitation data from the nearest meteorological station (Fukang) collected between 1951 and 2018 (Figure S1), we set up extreme precipitation treatments. Meanwhile, we obtained high-resolution precipitation data for our field sites from the Coupled Model Intercomparison Project 6 (CMIP6) climate projections data. These data were downloaded from the Climate Data Store [36], and we chose three shared socioeconomic pathway (SSP) scenarios: SSP1, SSP2, and SSP5 with greenhouse gas emissions that would potentially result in 1.9, 4.5 and 8.5 °C increases in global temperature, respectively, by 2100. SSP1, SSP2, and SSP5 represent an economic and social development path, a moderate development path, and a conventional development path, respectively [37]. In addition, we used the CAMS-CSM1-0 model to project changes in precipitation, and the model output consists of monthly precipitation in the future (to 2099). Finally, we divided the amount of precipitation into that of spring, summer, autumn, and winter, and calculated the coefficient of variation of precipitation every 10 years (Figure S2).

### 4.3. Experimental Design

The experiment was carried out in the experimental field of the Fukang Desert Research Station of the Chinese Academy of Sciences from July 2018 to July 2019. First, the sand (sand from the hinterland of the Gurbantunggut Desert) was sieved and put into a flowerpot (the outer diameter of flowerpot was 32 cm, the inner diameter was 31 cm, the height was 26 cm, and the lower diameter was 19 cm). Each flowerpot was filled with 15 kg sand and spread with a nylon net on the bottom of the flowerpot with drainage holes to facilitate ventilation and prevent the sand from leaking out. Then, the flowerpots were buried in the test field, with the edge of flowerpots about 5 cm above the ground. According to the annual precipitation data of the nearest city (Fukang) to the Gurbantunggut Desert from 1965 to 2018, the average annual precipitation was 274.19 mm, and the highest and lowest precipitation amounts were 452.70 and 145.90 mm, respectively. Meanwhile, the annual average precipitation was predicted to increase by 30% in northern China in the following 30 years [38,39,40], and the annual average precipitation fluctuations may reach +300% in extreme precipitation years (Figure S1a). Therefore, we defined the extreme precipitation threshold by calculating the relationship between the annual average precipitation, the annual highest precipitation, and the annual lowest precipitation in the Gurbantunggut Desert. Moreover, for the Gurbantunggut Desert, due to the stable snow in winter (the thickness of the snow can reach more than 30 cm), melting snow can keep the soil water content at a high level for nearly a month [35]. Therefore, considering the future precipitation trend and interannual precipitation fluctuations in the Gurbantunggut Desert, we set up five treatments (CK, control treatment; W1, +30% extreme precipitation; W2, +100% extreme precipitation; W3, +200% extreme precipitation; W4, maintenance of field capacity using a soil-weighing method every three days) with eight repetitions for each treatment. According to the daily precipitation data from a weather monitoring device (Caipos GmbH, Schillerstrasse Gleisdorf, Austria), we added water to flowerpots using a watering can after a precipitation event (including some snow). During the whole cycle of experiment, the CK, W1, W2, W3, and W4 treatments of SG plants were 108.90, 141.57, 217.80, 326.70, and 653.40 mm, respectively, and the CK, W1, W2, W3, and W4 treatments of AG plants were 186.70, 242.71, 373.40, 560.10, and 1120.20 mm, respectively. After seeds of *E. oxyrhynchum* were spread in the field, the seeds were sown evenly in the flowerpot (no seeds 1 cm from the edge), and the seeds were 2 cm away from the sandy soil surface. Sowing 100 seeds per flowerpot, 800 seeds, and eight flowerpots per treatment, a total of 8000 seeds and 80 flowerpots were needed (because there were only SG plants in 2018, the collected seeds were all from SG plants, and more than 91.00% were viable by using a 1% 2,3,5-triphenyltetrazolium chloride (TTC) solution). According to the precipitation data of each precipitation event from the weather station, we immediately increased precipitation after the end of the precipitation event.

### 4.4. Measurements and Sampling

The effects of extreme precipitation on phenology. Firstly, 10 SG and AG plants were selected for the phenology experiment under different extreme precipitation treatments. Secondly, we followed the methods of Lu et al. [18] during the phenology experiment and recorded the emergence date, flowering date, fruiting date, maturation date, and post-germination life span of different extreme precipitation treatments every three days until SG and AG plants died (from 1 July 2018 to 1 July 2019).

To find the effects of extreme precipitation on survival, we selected 10 SG and AG plants and recorded the number of survival plants at three-day intervals, except for when snow covered the flowerpots from November 2018 to March 2019, for each extreme precipitation treatment

To find the effects of extreme precipitation on organ size, according to the short life cycle of ephemeral plants and the phenology transition time, we measured organ size under different extreme precipitation treatments three times from 20 March to 20 May 2019, including plant height, leaf number, branch number, and seed number.

To find the effects of extreme precipitation on biomass accumulation and allocation, we measured organ size, including height, leaf number, branch number, and seed number when SG and AG plants reached the maturity stage under different extreme precipitation treatments. We then separated SG and AG plants according to roots, stems, leaves, and reproductive organs (flowers, fruits, and seeds), and the roots were carefully washed free of soil. Meanwhile, we harvested seeds from SG and AG plants at the time of maturity, i.e., when fruits were dry, yellow, and dehiscing. As a result of different extreme precipitation treatments, seeds matured at different times. Therefore, we collected the above- and below-ground organs of each plant until the seeds matured. We calculated the biomass of fruits (without seeds), leaves, stems, and roots (washed free of soil) of each plant after drying at 75 °C for 48 h using a Sartorius BS210S electronic balance (0.0001 g). Total biomass was calculated as the sum of roots, stems, leaves, and reproductive organ (flowers, fruits, and seeds) per plant. Finally, we calculated the biomass allocation of each part according to the biomass of each organ and individual biomass by the percentage of the total dry mass.

### 4.5. Statistical Analyses

Data from different extreme precipitation treatments were arcsine (percentage data) or log10 (other data) transformed before we conducted factor analyses to approximate the normal distribution and homogeneity of variance in order to fulfill the assumptions of two-way ANOVA. If the variance of transformed data was still not homogenous, treatment differences in these characteristics were assessed using the Kruskal–Wallis test. Then, all growth traits of SG and AG plants were analyzed as dependent variables with a two-way ANOVA under different extreme precipitation treatments. Emergence seasons and extreme precipitation were considered fixed effects, and all treatments terms were included. Tukey’s HSD test was performed for multiple comparisons to determine significant differences among SG and AG plants and different extreme precipitation treatments, and the results were considered significant when *p* < 0.05. Finally, we performed data analyses with the SPSS 13.0 statistical software package (SPSS Inc., Chicago, IL, USA) and drew all figures with Origin software 2015 (Origin Lab, Northampton, MA, USA).

## Figures and Tables

**Table 1:** Effects of extreme precipitation on phenology of SG and AG plants of *Erodium oxyrhynchum*.

Phenology	SG					AG				
	CK	W1	W2	W3	W4	CK	W1	W2	W3	W4
Emergence date	28 March	28 March	28 March	28 March	28 March	17 August	17 August	17 August	17 August	17 August
Leafing date	16 April	16 April	16 April	16 April	16 April	23 August	23 August	23 August	23 August	23 August
Flowering date	28 April	1 May	1 May	28 April	1 May	28 April	4 May	4 May	1 May	1 May
Fruiting date	16 May	16 May	16 May	16 May	16 May	16 May	13 May	13 May	13 May	10 May
Yellowing date	25 May	31 May	31 May	31 May	31 May	28 May	31 May	31 May	31 May	31 May

Note: Spring-germinated plants, SG; autumn-germinated plants, AG; CK, control treatment; W1, +30% extreme precipitation; W2, +100% extreme precipitation; W3, +200% extreme precipitation; W4, maintenance of field capacity by increasing precipitation every three days.

**Table 2:** Summary of a two-way ANOVA showing the effects of different emergence seasons and extreme precipitation on biomass accumulation and allocation of *Erodium oxyrhynchum*. P, extreme precipitation; S, emergence season; P*S, extreme precipitation × emergence season.

Plant Traits	Source of Variation	df	F	Sig.	Plant Traits	F	Sig.
Root biomass	S	1	8.752	<0.01	Root allocation	9.597	<0.01
	P	4	10.257	<0.01		9.386	<0.01
	P*S	4	13.788	<0.01		24.909	<0.01
Stem biomass	S	1	18.355	<0.01	Stem allocation	8.210	<0.01
	P	4	20.367	<0.01		58.476	<0.01
	P*S	4	13.480	<0.01		48.768	<0.01
Leaf biomass	S	1	6.761	<0.01	Leaf allocation	45.496	<0.01
	P	4	18.227	<0.01		21.335	<0.01
	P*S	4	15.183	<0.01		28.098	<0.01
Reproductive biomass	S	1	6.644	<0.01	Reproductive allocation	63.654	<0.01
	P	4	24.627	<0.01		30.297	<0.01
	P*S	4	16.082	<0.01		33.681	<0.01
Total biomass	S	1	13.473	<0.01			
	P	4	23.485	<0.01			
	P*S	4	16.065	<0.01			

## Data Availability

The data presented in this study are available in the article.

## Supplementary Materials

The following are available online at https://www.mdpi.com/article/10.3390/plants10081642/s1, Figure S1: Prediction of precipitation trend and coefficient of variation from spring, summer, autumn, and winter in the study area from 2020 to 2100. Figure S2: The average annual precipitation of the nearest weather station (Fukang) from 1965 to 2018 (a) and average daily temperature and precipitation from 1 July 2018 to 30 July 2019 (b) in the nearby city of Fukang.
