# Supplementary material for: Is Plant Life-History of Biseasonal Germination Consistent in Response to Extreme Precipitation?
Source: Plants (Basel). 2021 Aug 10;10(8):1642. doi: 10.3390/plants10081642 (PMC8402233; doi:10.3390/plants10081642)
Supplement: Supplementary file 1 [file plants-10-01642-s001.zip › plants-1280495-supplementary.pdf]

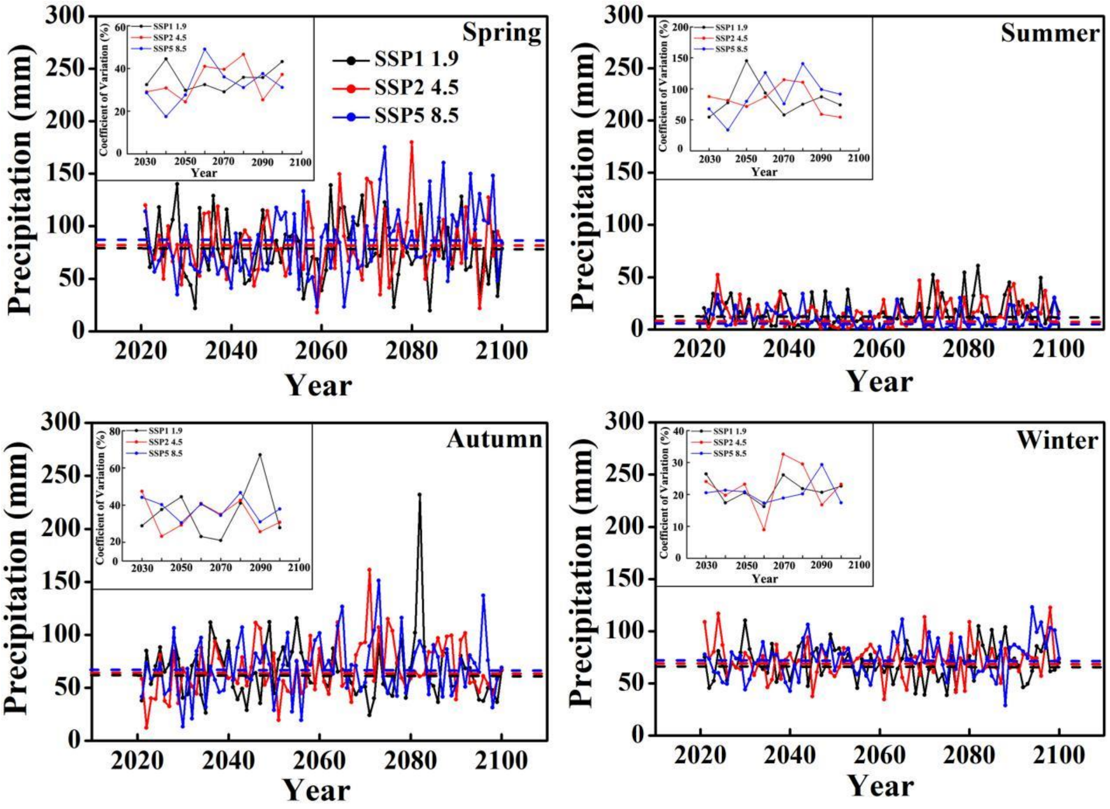

Figure S1. Prediction of precipitation trend and coefficient of variation from spring, summer, autumn, and winter in the study area from 2020 to 2100.

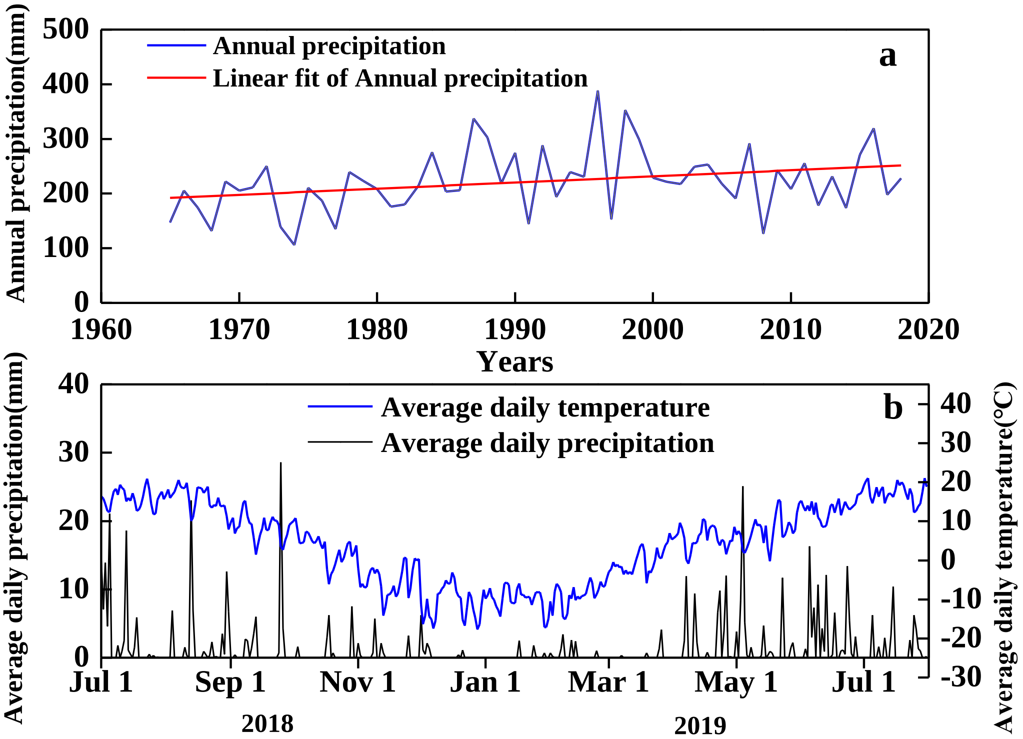

Figure S2. The average annual precipitation of the nearest weather station (Fukang) from 1965 to 2018 (a) and average daily temperature and precipitation from July 1, 2018 to July 30, 2019 (b) in the nearby city of Fukang.
